# The unconventional myosin CRINKLED and its mammalian orthologue MYO7A regulate caspases in their signalling roles

**DOI:** 10.1038/ncomms10972

**Published:** 2016-03-10

**Authors:** Mariam H. Orme, Gianmaria Liccardi, Nina Moderau, Rebecca Feltham, Sidonie Wicky-John, Tencho Tenev, Lior Aram, Rebecca Wilson, Katiuscia Bianchi, Otto Morris, Celia Monteiro Domingues, David Robertson, Meghana Tare, Alexander Wepf, David Williams, Andreas Bergmann, Matthias Gstaiger, Eli Arama, Paulo S. Ribeiro, Pascal Meier

**Affiliations:** 1 Chester Beatty Laboratories, The Breast Cancer Now Toby Robins Research Centre, Institute of Cancer Research, Mary-Jean Mitchell Green Building, 237 Fulham Road, London SW3 6JB, UK; 2 John Vane Science Centre, Centre for Tumour Biology, Barts Cancer Institute, Queen Mary University of London, Charterhouse Square, London EC1M 6BQ, UK; 3 Department of Molecular Genetics, Weizmann Institute of Science, 1307 Meyer Building, Rehovot 76100, Israel; 4 University of Massachusetts Medical School, 364 Plantation Street, RB Suite 419, Worcester Massachusetts 01605, USA; 5 ETH, Institute for Molecular Systems Biology, Wolfgang Pauli-Strasse 16, CH-8093 Zurich, Switzerland; 6 UCLA David Geffen School of Medicine, 200 Stein Plaza, Los Angeles, California 90095-7008, USA

## Abstract

Caspases provide vital links in non-apoptotic regulatory networks controlling inflammation, compensatory proliferation, morphology and cell migration. How caspases are activated under non-apoptotic conditions and process a selective set of substrates without killing the cell remain enigmatic. Here we find that the *Drosophila* unconventional myosin CRINKLED (CK) selectively interacts with the initiator caspase DRONC and regulates some of its non-apoptotic functions. Loss of CK in the arista, border cells or proneural clusters of the wing imaginal discs affects DRONC-dependent patterning. Our data indicate that CK acts as substrate adaptor, recruiting SHAGGY46/GSK3-β to DRONC, thereby facilitating caspase-mediated cleavage and localized modulation of kinase activity. Similarly, the mammalian CK counterpart, MYO7A, binds to and impinges on CASPASE-8, revealing a new regulatory axis affecting receptor interacting protein kinase-1 (RIPK1)>CASPASE-8 signalling. Together, our results expose a conserved role for unconventional myosins in transducing caspase-dependent regulation of kinases, allowing them to take part in specific signalling events.

## Abstract (web summary)

In addition to their role in apoptosis, caspases are also involved in mediating non-apoptotic events. Here the authors show that the *Drosophila* myosin family member CRINKLED and its mammalian counterpart act as substrate adaptor that facilitate caspase-mediated cleavage and localised kinase activity.

## Graphical abstract

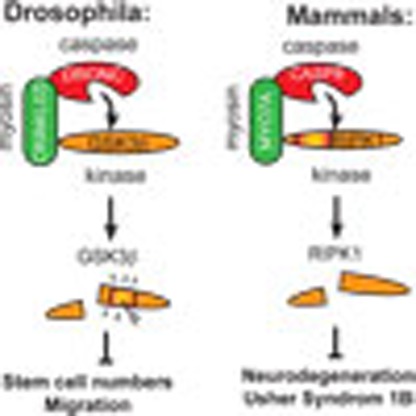

Deregulation of caspases forms the basis of many human disease pathogeneses including neurodegeneration and cancer^1^. Although caspases have been extensively studied as initiators, executioners or regulators of cell death mediated by apoptosis, pyroptosis, necroptosis or autophagy^2^, it is clear that caspases actively regulate animal development and the defense of homeostasis through both cell death-dependent and -independent functions^3^,^4^,^5^. Caspase activation requires the recruitment of initiator caspases into macromolecular protein complexes that mediate the activation of initiator caspases through proximity-induced dimerization. Activation of initiator caspases depends on the engagement of platforms such as the death-inducing signalling complex, complex-II or ripoptosome for CASPASE-8 (CASP8) or CASP10, the apoptosome for CASP9 and the inflammasome for CASP-1 or -11 (ref. 6). These platforms integrate cellular signals and recruit initiator caspases via their death-fold domain, which results in the dimerization of the initiator caspases and formation of an active enzyme^6^. An important outstanding question is how caspases can be activated to mediate non-apoptotic events without killing the cell. Hypotheses that have been suggested include temporal restriction of activity and amplitude modulation (see ref. 3); however, it is not clear how general these modes of regulation are.

By studying how caspases take part in non-apoptotic signalling, we unexpectedly discovered an evolutionary conserved principle of caspase-mediated control of cellular processes. We find that in both *Drosophila* and mammals, an unconventional myosin is essential for caspase-mediated regulation of kinases. Our data demonstrate that the *Drosophila* myosin family member CRINKLED (CK) and its mammalian counterpart Myosin VIIA (MYO7A) act as substrate adaptor for kinases, thereby facilitating caspase-mediated cleavage and localized modulation of kinase activity. In mammals, this results in inactivation of RIPK1 and suppression of CASP8. In the absence of MYO7A, CASP8-mediated cleavage and inactivation of RIPK1 is less effective. This has important implications, because mutations in MYO7A cause Usher syndrome 1B—an autosomal recessive disorder characterized by bilateral sensorineural hearing loss and blindness due to retinitis pigmentosa. Despite intense investigation, the mechanisms by which loss of MYO7A results in deafness and blindness are poorly understood. Our finding that MYO7A interacts with the initiator CASP8 and dampens its activation may help to explain why patients with mutations in MYO7A suffer progressive loss of sensory neurons. Given that RIPK1 and CASP8 take part in the defense of homeostasis downstream of many cytokine receptors, it is plausible that inflammatory signals contribute to the onset and progression of retinitis pigmentosa in patients with MYO7A mutations due to aberrant activation of RIPK1-dependent cell death.

## Results

### CK modulates DRONC-dependent phenotypes

**Figure 1:**
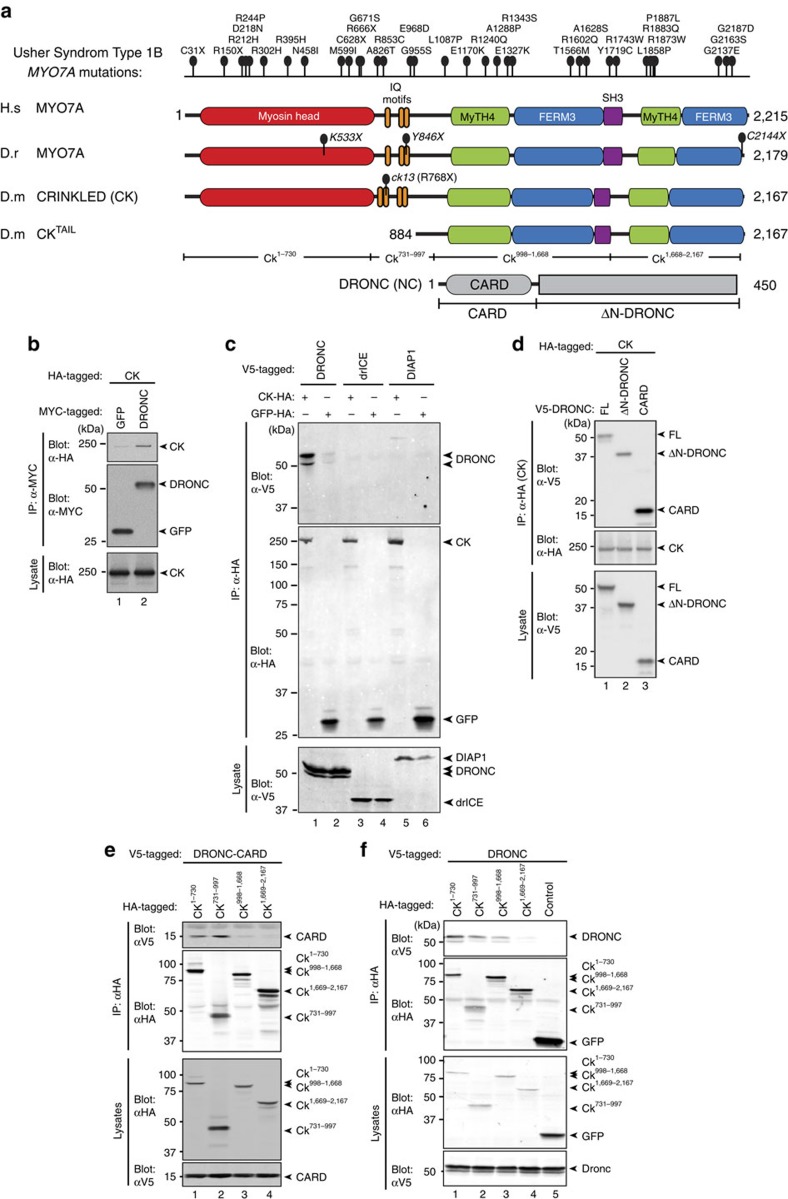
CK selectively interacts with DRONC. (**a**) Schematic representation of MYO7A from human (*Homo sapiens*), zebrafish (*Danio rerio*) and *Drosophila* (*D. melanogaster*). Top: more than 120 mutations in the *MYO7A* gene have been identified in patients with Usher syndrome type 1B (http://mutdb.org/products/search). Indicated are 34 selected mutations in human MYO7A and the respective *mariner* mutations of zebrafish MYO7A. The loss-of-function mutation in CK (*ck*^*13*^) is indicated. (**b**) MYC-GFP or MYC-DRONC was co-expressed with HA-CK in *Drosophila* S2 cells. α-MYC-immunoprecipitation was performed and CK interaction was assessed via immunoblotting. (**c**) V5-DRONC, V5-drICE or V5-DIAP1 was co-expressed with HA-CK or HA-GFP in S2 cells. α-HA immunoprecipitation was performed and binding to CK or GFP was determined by immunoblotting. (**d**) V5-tagged full-length DRONC, ΔN DRONC (see scheme in **a**) or DRONC CARD (CARD) were co-expressed with HA-CK. α-HA immunoprecipitation was performed and binding to CK was determined by immunoblotting. (**e**) V5-CARD of DRONC was co-expressed with the indicated HA-tagged fragments of CK. α-HA immunoprecipitation was performed and binding to CK was determined by immunoblotting. (**f**) V5-tagged full-length DRONC was co-expressed with the indicated HA-tagged fragments of CK. α-HA immunoprecipitation was performed and binding to CK was determined by immunoblotting.

To elucidate how caspases are regulated in their apoptotic and non-apoptotic roles, we set out to identify new binding partners of the *Drosophila* initiator caspase DRONC. To this end, an HA_3*x*_-tagged form of DRONC was stably expressed in *Drosophila* Schneider cells (S2). DRONC protein complexes were isolated via large-scale affinity purification from *Drosophila* S2 cells using α-HA resin followed by mass spectrometric analysis. As controls, we used cells stably expressing HA_3*x*_-tagged forms of green fluorescent protein (GFP), DARK, DIAP1, DIAP2, DREDD, dTAK1, dTAB2, RELISH, KENNY and IRD5. Using DRONC as an affinity reagent, we identified a total of 89 proteins with a minimum score of two unique peptides (Supplementary Table 1). CK, the *Drosophila* orthologue of mammalian non-muscle MYO7A selectively co-purified with DRONC (Fig. 1a–c). Although we identified five unique CK-derived peptides in DRONC immunoprecipitates, no such peptides were identified in control immunoprecipitates, highlighting the selectivity of the CK–DRONC interaction (Supplementary Table 1). Reciprocal co-immunoprecipitation assays demonstrated that CK specifically associated with DRONC, while it did not interact with GFP, DIAP1 or drICE (Fig. 1b,c). To identify the regions of DRONC and CK that are required for their interaction, we tested several DRONC and CK fragments for their ability to bind to CK or DRONC, respectively. We found that CK preferentially bound to the CARD-containing pro-domain of DRONC (Fig. 1d). In addition, CK also weakly interacted with the p20-p10 region of DRONC, suggesting that the interaction between CK and DRONC is mediated by multiple contact points. The CARD domain of DRONC interacted with the amino-terminal portion of CK (CK^1–997^) encompassing the myosin head and IQ motifs (Fig. 1e), whereas the caspase domain of DRONC (p20-p10) region associated with the central region of CK (CK^998–1,668^) (Fig. 1f).

**Figure 2:**
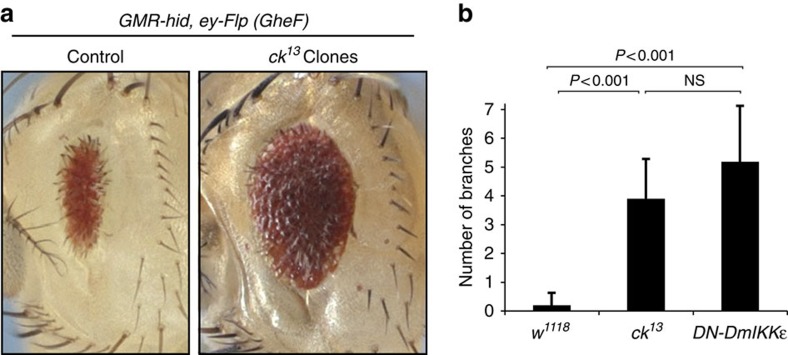
Loss of CK modulates DRONC-dependent phenotypes. (**a**) A loss-of-function mutation in CK, *ck*^*13*^, suppresses the *GMR-hid*-induced eye phenotype. The left panel depicts the unsuppressed *GMR-hid,ey-Flp* (*GheF*) eye ablation phenotype (*GMR-hid*, *ey-Flp/+* or *Y; FRT40A/P[ubi-GFP]*, *FRT40A*). Suppression of the *GheF* phenotype in *ey-Flp/FRT*-induced clones of *ck*^*13*^ (*GMR-hid*, *ey-Flp/+* or *Y;ck*^*13*^, *FRT40A/P[ubi-GFP]*, *FRT40A*). This *ck* allele gives rise to strong suppression of *GMR-hid* and is a loss-of-function mutant. (**b**) Mutation in *ck* such as expression of DN DmIKK*ɛ* causes ectopic branching of the arista. Indicated is the number of branch points per lateral of the arista. First bar: *w*^*1118*^ (*n*=10). Second bar*: ck*^*13*^ (*n*=10). Third bar: *Ap-Gal4/UAS-DmIKKɛ*^*G250D*^ (*n*=11). Error bars represent s.d. *P*-values are <0.001 with respect to the control. NS indicates not significant. *P*-values were calculated using an unpaired, two-tailed *t*-test.

CK represents an unconventional, non-muscle myosin that is highly conserved from flies to man, sharing 73% amino acid sequence similarity to its human counterpart MYO7A (Fig. 1a). MYO7A is expressed in numerous epithelial cell types, suggesting a role in multiple cellular processes. Mutations of MYO7A in humans cause Usher syndrome 1B (Fig. 1a), an autosomal recessive disorder characterized by bilateral sensorineural hearing loss and blindness due to retinitis pigmentosa^7^. Despite intense investigation, the mechanisms by which loss of MYO7A results in deafness and blindness are poorly understood^8^. In *Drosophila*, a loss-of-function mutation in CK (*ck*^*13*^) causes early embryonic lethality^9^, indicating that CK is indispensable for normal development. To investigate the functional significance of the interaction between CK and DRONC, we examined the requirement of CK in head involution defective (HID)-mediated eye ablation (Fig. 2a). Expression of the inhibitor of apoptosis (IAP) antagonist HID in the eye activates DRONC-dependent cell death, resulting in a small eye phenotype that is sensitive to the dosage of regulatory genes that control components of the apoptotic machinery^10^,^11^,^12^,^13^,^14^,^15^,^16^,^17^. Using the previously described *GheF* (*GMR-hid ey-FLP*) method^18^, we found that eye-specific loss of CK (*ck*^*13*^ homozygous mutant clones in the eye) significantly protected from the *GMR-hid* induced eye-ablation phenotype (Fig. 2a). Importantly, loss of *ck* function did not affect expression of HID protein, ruling out the possibility that *ck*^*13*^ suppresses the HID eye phenotype by altering HID protein levels (Supplementary Fig. 1a–d). Similar to genetic deletion of *ck*, RNA interference (RNAi)-mediated knockdown of *ck* also suppressed HID- and REAPER (RPR) killing in the eye (Supplementary Fig. 1e–h). Together, our data indicate that CK binds to DRONC and can contribute to DRONC-dependent phenotypes, such as efficient induction of cell death in response to high-level expression of the IAP antagonists HID and RPR.

To study the physiological role of CK in regulating DRONC, we investigated various paradigms of DRONC-mediated apoptotic and non-apoptotic signalling events. Rare adult ‘escapers' of *ck*^*13*^ mutant flies that occasionally emerge exhibit aristae, which are more highly branched than normal^9^ (Fig. 2b and Supplementary Fig. 1i). Interestingly, branching of the *Drosophila* arista is regulated by DRONC^19^,^20^, an initiator caspase that is controlled by DIAP1 (ref. 11). Accordingly, surviving flies carrying the semi-lethal nonsense mutations *Dronc*^*I24*^ or *Dronc*^*I29*^ display extra aristal branches compared with wild-type controls^19^. Likewise, suppression of DRONC activity via RNAi-mediated inactivation of *Drosophila Ikk*, *DmIKKɛ* (also known as *Ik2*), which results in an increase in DIAP1 levels and lower DRONC activity^20^, causes excessive branching of the arista (Fig. 2b) (refs 9, 21). Ectopic branching is also observed in *hid* mutants^22^. Although failure to activate DRONC results in ectopic branching, a branchless or thread-like phenotype of the arista is observed in flies that carry the DIAP1 loss-of-function mutant *thread*^*1 22*^. This indicates that controlled activation of DRONC is required for proper arista morphogenesis. The notion that CK interacts with DRONC, and *ck*^*13*^ and *Dronc* mutants display extra branches of the arista strongly suggests that CK regulates DRONC in the arista.

Next, we investigated the role of CK in regulating developmentally controlled or stress-induced apoptosis. A large amount of naturally occurring cell death is observed anterior to the morphogenetic furrow in the third-instar larval eye disc^23^ and this cell death was not suppressed in *ck*^*13*^ mutant clones (Supplementary Fig. 2a,b). We also examined pupal eye development, during which apoptosis is required to remove excess interommatidial cells. We found that the number of interommatidial cells was not affected in *ck*^*13*^-null clones (Supplementary Fig. 2c,d) (two-tailed *t*-test: *P*=0.38). Loss of *ck* also did not affect X-irradiation-induced DRONC activation in the wing discs of third-instar larvae (Supplementary Fig. 2e). Together, these data indicate that CK does not regulate DRONC in its apoptotic role in the fly. It is possible that although strong expression of HID and RPR promote cell death through CK-assisted activation of DRONC, CK-mediated DRONC activation might not be a rate-limiting factor during naturally occurring cell death scenarios where apoptosis is executed through the concerted action of mitochondrial-dependent and -independent mechanisms^13^,^24^,^25^,^26^.

### CK is required to suppress extra macrochaete in the scutellum

**Figure 3:**
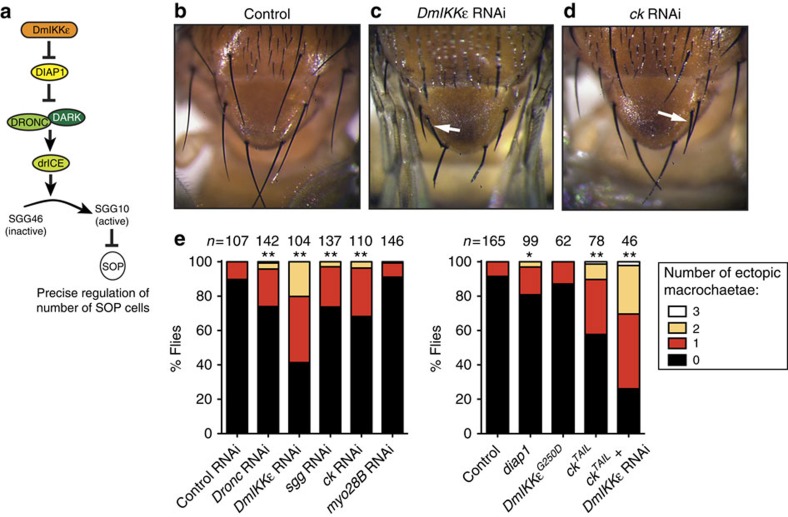
CK but not its close homologue MYO28B is required for DRONC activation and the suppression of extra macrochaete in the scutellum. (**a**) Schematic diagram depicting the regulation of normal SOP cell number. (**b**–**d**) Light microscope images of macrochaete in the scutellum of the adult notum. White arrows indicate ectopic macrochaete. Genotypes: (**b**) *Canton S.* (**c**) *UAS-DmIKKɛ-IR/sca-Gal4*. (**d**) *sca-Gal4/+; UAS-ck-IR/+*. (**e**) Quantification of the number of ectopic macrochaete on the notum of adult flies of the indicated genotypes. *P-value <0.05 with respect to the control and **P-value <0.005 with respect to the control. *P*-values were calculated using Fisher's exact test. Genotypes: Control RNAi: *UAS-GFP-IR/sca-Gal4*. *Dronc* RNAi: *UAS-Dronc-IR/sca-Gal4*. *DmIKKɛ* RNAi: as in **c**. *sgg* RNAi: *UAS-sgg-IR/sca-Gal4. ck* RNAi as in **d**. *myo28B* RNAi: *UAS-myo28B-IR/sca-Gal4*. Control: *UAS-GFP:LacZ-nls/sca-Gal4*. *diap1*: *UAS-diap1/sca-Gal4*. *DmIKKɛ*^*G250D*^: *sca-Gal4/+; UAS-DmIKKɛ*^*G250D*^*/+*. *ck*^*TAIL*^: *sca-Gal4/+; UAS-ck*^*TAIL*^/+. *ck*^*TAIL*^+*DmIKKɛ RNAi*: *UAS-DmIKKɛ-IR/sca-Gal4; UAS-ck*^*TAIL*^/+.

Caspase activity without cell death controls various physiological processes, including cell differentiation and cell migration^3^,^4^,^5^; however, the regulatory mechanisms of caspase activity in this context are largely unknown. With this in mind, we examined the effects of CK loss on caspase-mediated signalling events, independent of cell death. The development of sensory organ precursor (SOP) cells and the correct number of macrochaete (large bristles) on the scutellum of the notum are determined through a non-apoptotic signalling function of DRONC^21^,^27^ (Fig. 3a). Non-apoptotic DRONC activation is achieved through DmIKKɛ-mediated depletion of DIAP1 protein levels in cells of the proneural cluster that gives rise to SOPs. Partial depletion of DIAP1 causes caspase activation and caspase-mediated cleavage of the inactive GSK3β precursor SHAGGY (SGG) 46 protein. This cleavage converts it to the active kinase SGG10, which contributes to SOP cell specification^21^,^27^ (Fig. 3a), allowing the formation of precisely four macrochaete on the scutellum. However, ectopic macrochaete develop if caspase activation or activity is suppressed^21^,^27^. Thus, we examined whether loss of *ck* phenocopies depletion of *Dronc*, *sgg* and *DmIKKɛ* in specifying numbers of macrochaete in the scutellum. Knockdown of *ck* in the scutellum, using the *scabrous-GAL4* (*Sca-GAL4*) driver, resulted in the appearance of extra macrochaete in 32% of flies (*n*=110; Fig. 3b,d,e). This was reminiscent of the effects of *Dronc* RNAi (26%, *n*=142), *sgg* RNAi (26%, *n*=137) and *DmIKKɛ* RNAi (59%, *n*=104) (Fig. 3b,c,e). Interestingly, depletion of *ck* phenocopies expression of a non-cleavable form of SGG46 (*sgg46*^*D235G/D300G* 27^, thereafter referred to as *sgg46*^*D>G*^), which causes the appearance of ectopic macrochaete in ∼19.3% of animals^27^. This suggests that SGG46 fails to be cleaved in the absence of *ck*. Although knockdown of *ck* resulted in extra macrochaete, RNAi against the closely related gene *myo28B* (the orthologue of mammalian *Myo7B*) did not lead to the formation of extra machrochaete (*n*=146) (Fig. 3e). This suggests that CK, but not MYO28B, is required for DRONC activation in proneural clusters.

Consistent with the phenotype obtained by *ck* RNAi, expression of a dominant-negative (DN) form of CK (CK^TAIL^ (ref. 28)) also resulted in ectopic macrochaete in ∼42% of flies (*n*=78), compared with 8% in controls (*n*=165). Expression of CK^TAIL^ enhanced the ectopic macroachaete phenotype caused by *DmIKKɛ* RNAi (Fig. 3e). These observations suggest that CK regulates DRONC in its non-apoptotic role in proneural clusters, to determine the correct number of SOP cells.

**Figure 4:**
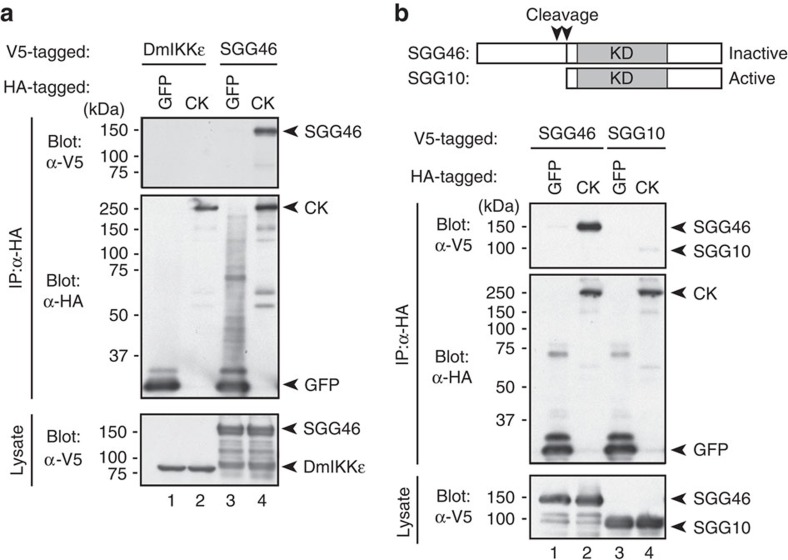
CK interacts with SGG46. (**a**) V5-DmIKKɛ or V5-SGG46 was co-expressed with HA-GFP or HA-CK in *Drosophila* S2 cells. α-HA-immunoprecipitation was performed and CK interaction was assessed via immunoblotting. (**b**) V5-SGG46 or V5-SGG10 was co-expressed with HA-GFP or HA-CK in *Drosophila* S2 cells. α-HA-immunoprecipitation was performed and CK interaction was assessed via immunoblotting. Top panel: schematic diagram indicating the drICE cleavage sites on SGG. KD, kinase domain.

To gain insight into the mechanism by which CK regulates the number of macrochaete, we investigated whether CK might function as a substrate adaptor linking DRONC to SGG46 and facilitating its conversion to SGG10. Binding assays revealed that CK specifically co-purified SGG46 from cellular extracts, whereas it did not associate with DmIKKɛ under the same conditions (Fig. 4a). Intriguingly, CK bound exclusively to the inactive GSK3β precursor SGG46 and did not associate with SGG10 (Fig. 4b). Given that CK interacts with both DRONC and the precursor form of SGG46, and that loss of *ck* phenocopies loss of *Dark*, *Dronc* and *sgg* or expression of SGG46^D>G^, our data suggest a mechanism whereby CK acts as a substrate adaptor bringing SGG46 in close proximity to the caspase, thereby facilitating cleavage and localized activation of SGG46. According to this scenario, following caspase-mediated cleavage, active SGG10 is then released to regulate SOP cell specification. To gain a better understanding why CK^TAIL^ acts as a DN mutant, we tested the ability of CK^TAIL^ to bind to DRONC. As shown in Supplementary Fig. 3a, CK^TAIL^ bound to DRONC as efficiently as wild-type CK. As CK helps to recruit substrates (such as SGG46) to DRONC, it is likely to be that CK^TAIL^ fails to associate with caspase substrates. Thus, CK^TAIL^ may function as DN, because it prevents the recruitment of caspase substrates to DRONC. Validation of how CK modulates DRONC signalling will ultimately require the reconstitution of stable DARK/DRONC/drICE/CK/SGG46 protein complexes. Attempts to reconstitute this complex *in vitro* have been hampered by difficulties in producing recombinant CK due to its sheer size (2,176 amino acids).

### DN forms of CK rescue the *Rac* ^*N17*^ migration defect

**Figure 5:**
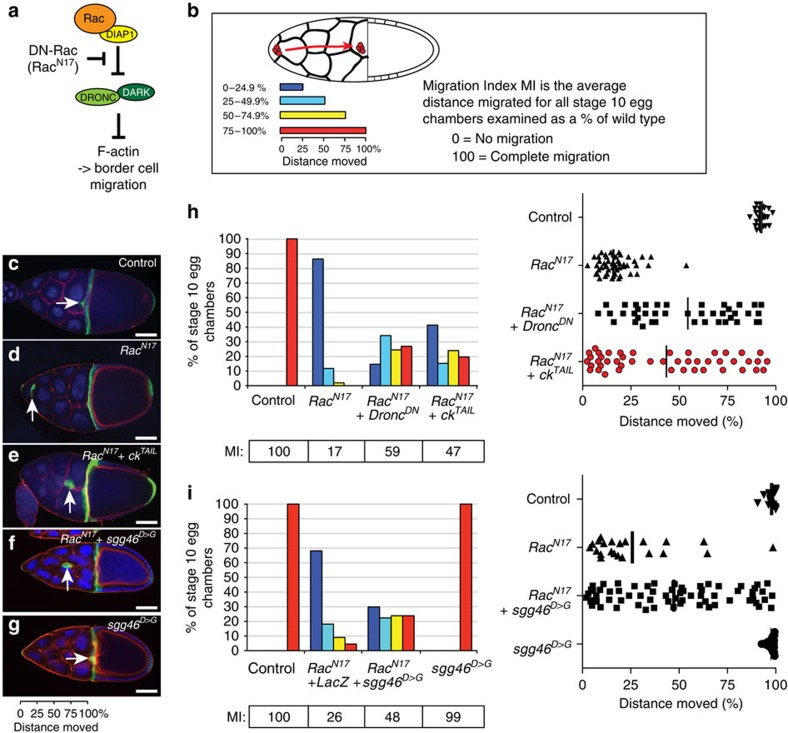
Expression of DN forms of CK rescues the RAC^N17^ migration defect. (**a**) Schematic diagram depicting Rac-mediated regulation of border cell migration. (**b**) Schematic drawing of a stage-10 egg chamber. Border cells and their moving trajectory are indicated in red. (**c**–**g**) Confocal microscope images of stage-10 egg chambers of the indicated genotypes. Border cell clusters are indicated by arrows. Blue: DAPI (4,6-diamidino-2-phenylindole). Green: GFP, indicating border cells. Red: Phalloidin. Scale bars, 50 μm. Genotypes: (**c**) *slbo-Gal4*, *UAS-GFP*/*+*. (**d**) *slbo-Gal4*, *UAS-GFP*/*+*; *UAS-Rac*^*N17*^/*+*. (**e**) *slbo-Gal4*, *UAS-GFP*/*+*; *UAS-Rac*^*N17*^/*UAS-ck*^*TAIL*^. (**f**) *slbo-Gal4*, *UAS-GFP*/*UAS-MYCsgg46*^*D235G/D300G*^; *UAS-Rac*^*N17*^/+. (**g**) *slbo-Gal4*, *UAS-GFP*/*UAS-MYCsgg46*^*D235G/D300G*^; *+*/*+.* (**h**,**i**) Rescue of border cell migration defects. Graphs showing the distance moved by border cells in egg chambers from flies of the indicated genotypes. The data are plotted using a bar diagram (left) or scatter plot (right). Bars in the scatter plot represent the median values. Genotypes: control: as in **c**. *Rac*^*N17*^. (**h**): as in **d**. *Rac*^*N17*^+*LacZ*. (**i**) *slbo-Gal4*, *UAS-GFP*/*UAS-LacZ*;*UAS-Rac*^*N17*^/+. *Rac*^*N17*^+*Dronc*^*DN*^: *slbo-Gal4*, *UAS-GFP*/*+*; *UAS-Rac*^*N17*^/*UAS-Dronc*^*C>A*^. *Rac*^*N17*^+*ck*^*TAIL*^: as in **e**. *Rac*^*N17*^+*sgg46*^*D>G*^: as in **f**. *sgg46*^*D>G*^: as in **g**.

The migration of border cells in the developing egg chamber is another non-apoptotic developmental process in which DmIKKɛ and DRONC have been implicated^29^. Border cell migration provides a simple model to study how cells that originate within an epithelial monolayer subsequently become migratory. Border cells undergo ‘collective cell migration' from the anterior pole of the developing egg chamber towards the oocyte, reaching their destination by stage 10 of oocyte development. The collective migration of border cells is dependent on the activity of the small GTPase RAC. Expression of a DN form of RAC (RAC^N17^) almost entirely blocked migration of the border cell cluster^29^ (Fig. 5a,b,d,f,h,i). Importantly, this migration defect can be rescued through suppression of DRONC activity, either by mutations affecting DARK, expression of a DN form of DRONC (DRONC^DN^) or expression of DIAP1 (ref. 29). The role for DRONC in negatively regulating RAC-mediated cell motility represents an apoptosis-independent function of DRONC. Given that the migratory phenotype of RAC^N17^-expressing border cells is sensitive to DRONC activity, we assessed whether CK is required for DRONC activation in border cells. Normal border cells complete migration to 100% at stage 10 (Fig. 5c,h,i). In contrast, *slbo-GAL4*-driven expression of *UAS-Rac*^*N17*^ resulted in severe migration defects in which none of the border cell clusters reached their final destination (Fig. 5d,h,i). Less than 2% of RAC^N17^-expressing egg chambers had border cell clusters that reached halfway to their presumptive destination. Importantly, expression of CK^TAIL^ suppressed the RAC^N17^ migration defect significantly, with 43.5% of egg chambers having border cells that migrated at least halfway to the oocyte (Fig. 5e,h). The ability of CK^TAIL^ to rescue the RAC^N17^ was comparable to that of DRONC^DN^, expression of which resulted in 51.2% of egg chambers having border cells that migrated at least halfway to the oocyte (Fig. 5h). Although no RAC^N17^ egg chambers had border cells that migrated beyond 75% of the total distance, expression of DN forms of CK and DRONC rescued the *Rac*^*N17*^ phenotype, allowing border cells in 19.6% and 26.8%, respectively, of the egg chambers to migrate further than 75% of their distance. Given that CK^TAIL^ phenocopies DRONC^DN^ in rescuing the RAC^N17^ migration phenotype, our data further demonstrate a functional interaction between CK and DRONC, and are consistent with the notion that CK contributes to DRONC-mediated non-apoptotic signalling.

Next, we addressed whether CK affects caspase signalling through a common mechanism in these multiple models of caspase function. During SOP specification, CK seems to function as substrate adaptor that helps to bridge DRONC and SGG46. To test the role of SGG46 cleavage in border cell migration, we expressed SGG46^D>G^ that cannot be cleaved^27^ (Fig. 5f,g,i). If the RAC^N17^ migration defect was mediated by DRONC-dependent cleavage of SGG46, the prediction is that a non-cleavable form of SGG46 would phenocopy DRONC^DN^ and CK^TAIL^, and rescue the RAC^N17^ block in migration. Indeed, expression of SGG46^D>G^ rescued the RAC^N17^ migration phenotype (Fig. 5f,g,i). Importantly, the ability of SGG46^D>G^ to rescue the RAC^N17^ phenotype was comparable to the effect of DRONC^DN^ and CK^TAIL^ (Fig. 5 and also compare Fig. 5h with Fig. 5i). This also demonstrates that cleavage of a single substrate is sufficient to mediate the RAC^N17^ phenotype. Given that SGG46^D>G^ phenocopies CK^TAIL^ and DRONC^DN^ in rescuing the RAC^N17^ migration phenotype, our data are consistent with the notion that RAC^N17^ drives CK-mediated activation of DRONC, which results in caspase-dependent cleavage and activation of SGG46. Activation of SGG subsequently suppresses border cell migration, most probably by influencing the dynamics of actin cytoskeleton, microtubule and adhesion turnover.

We further investigated the role of CK in regulating two other caspase-dependent signalling pathways, *Drosophila* innate immune signalling and spermatid individualization. We found that CK specifically interacted with the death-domain-containing protein immune defiency (IMD) and ameliorated IMD signalling following septic injury with the Gram-negative bacteria *Ecc15* (Supplementary Fig. 3b–d). This is consistent with an earlier report implicating CK as a putative negative regulator of IMD signalling^30^. Although CK regulates initiator caspases in their non-apoptotic roles in border cells, SOP and arista, CK appeared to play no rate-limiting role in DRONC-mediated individualization of spermatids (Supplementary Fig. 3e). Together, our data demonstrate that CK regulates caspases in some but not all signalling aspects. Quite possibly, SGG46 is only involved in border cells, SOP, arista and fat body cells (for IMD signalling), but not in spermatids.

### MYO7A binds to CASP8 in a RIPK1-dependent manner

**Figure 6:**
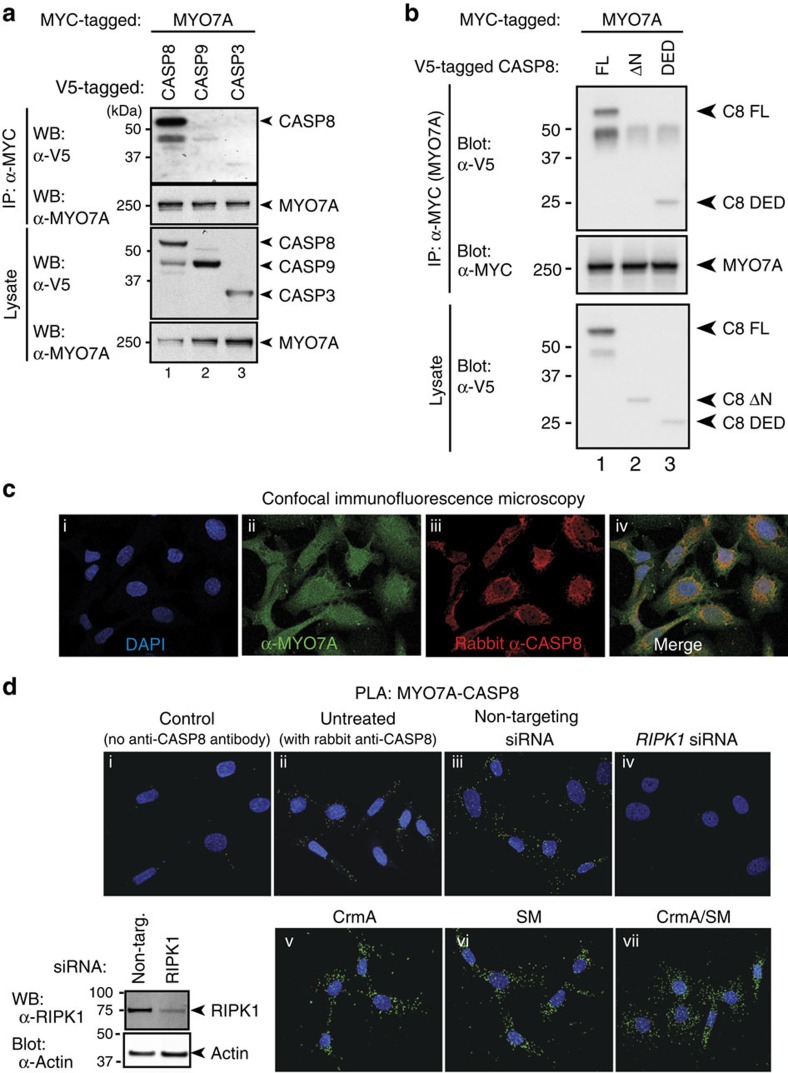
MYO7A selectively binds to CASP8. (**a**) MYC-MYO7A was co-expressed with V5-CASP8, V5-CASP9 or V5-CASP3 in 293T cells. α-MYC-immunoprecipitation was performed and MYO7A interaction was assessed via immunoblotting. (**b**) The indicated proteins were co-expressed in 293T cells and the interaction between MYO7A and full-length or deletion mutants of CASP8 was assessed by immunoprecipitation followed by immunoblotting. (**c**) Confocal microscopy images of SKOV3 cells showing co-localization of MYO7A (green) and CASP8 (red) using the indicated antibodies. Nuclei stained with DAPI (4,6-diamidino-2-phenylindole; blue). (**d**) Representative confocal microscopy images of proximity ligation assay (PLA) using α-MYO7A and either no α-CASP8 antibody (i) or rabbit α-CASP8 antibody (i–vii) in SKOV3 cells. PLA signals are in green and nuclei are stained with DAPI (blue). PLA between MYO7A and CASP8 on siRNA knockdown of control (non-targeting oligos) (iii) or RIPK1 (iv). Immunoblot analysis of RIPK1 and ACTIN following siRNA knockdown of control or *Ripk1*. PLA between MYO7A and CASP8 in cells stably expressing CrmA (v), on treatment with 100 nM SM for 5 h (vi), or in response to SM/CrmA treatment (vii).

Human MYO7A shares 73% amino acid sequence similarity to *Drosophila* CK. Mutations in *MYO7A* cause Usher syndrome type 1B, a disease characterized by the combination of sensorineural hearing loss and visual impairment termed retinitis pigmentosa^7^. Previous studies of the mutant zebra fish *mariner*, which carries a mutation in *myo7aa* (the zebra fish homologue of MYO7A), revealed mild endoplasmic reticulum (ER) stress and sensory neuron apoptosis^31^. However, the mechanism through which such *MYO7A* alleles render cells susceptible to apoptosis remains ill defined. Given that CK directly interacts with the initiator caspase DRONC, we assessed whether MYO7A might bind and regulate caspases. To this end, we tested the ability of mammalian MYO7A to associate with CASP8, CASP9 and the effector CASP3. Intriguingly, we found that MYO7A selectively bound to CASP8, whereas it failed to associate with CASP3 or CASP9 under the same experimental conditions (Fig. 6a). The binding of CASP8 to MYO7A was mediated by the death effector domain (DED) of CASP8, as this domain in isolation specifically interacted with MYO7A (Fig. 6b). Consistently, deletion of the DED (ΔN-CASP8) completely abrogated the binding of CASP8 to MYO7A, indicating that MYO7A only binds to pro-CASP8.

To investigate the localization and interaction of endogenous MYO7A and CASP8, we performed immunofluorescence confocal microscopy. Staining with MYO7A- and CASP8-specific antibodies revealed that the expression of endogenous MYO7A closely overlapped with that of CASP8 (Fig. 6c and Supplementary Fig. 4a). Two different α-CASP8 antibodies, when combined with α-MYO7A antibodies, provided identical results (compare Fig. 6c with Supplementary Fig. 4a).

To visualize MYO7A/CASP8 protein interaction in their native state in intact cells, we applied *in situ* proximity ligation assay (PLA)^32^ with a combination of antibodies that generate a localized, discrete signal only when CASP8 and MYO7A are in close proximity, that is, in a complex. Using specific primary antibodies against CASP8 and MYO7A, which in turn were recognized by oligonucleotide-coupled secondary antibodies, we obtained discrete proximity labelling of CASP8 and MYO7A, confirming their close proximity (Fig. 6d). Virtually, the same result was obtained using two different primary α-CASP8 antibodies (Fig. 6d and Supplementary Fig. 4b). To control for nonspecific binding between the various secondary antibodies, we added only one or no primary antibody to the reaction and observed no signal (Fig. 6d). Intriguingly, MYO7A/CASP8 proximity labelling was entirely dependent on the presence of RIPK1. Accordingly, depletion of *Ripk1* by RNAi completely abolished CASP8/MYO7A proximity signals (Fig. 6d, compare d(ii) and d(iii) with d(iv)). This result also demonstrates that the observed proximity signal is not due to nonspecific binding of the primary antibodies. The requirement of RIPK1 for the generation of CASP8/MYO7A proximity signals strongly suggests that CASP8 and MYO7A interact with one another within RIPK1-based protein complexes such as the ripoptosome.

### MYO7A suppresses ripoptosome formation

**Figure 7:**
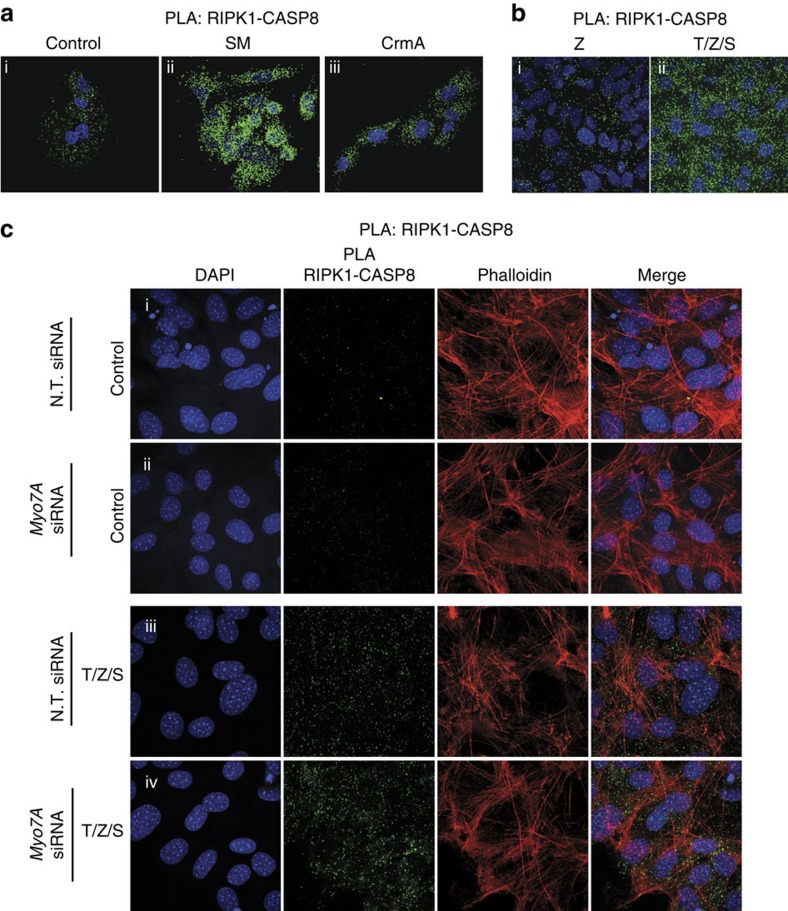
MYO7A suppresses ripoptosome formation. (**a**) Proximity ligation assay (PLA) between RIPK1 and CASP8 in SKOV3 cells on control treatment (i), 100 nM SM for 5 h or in cells stably expressing CrmA (iii). (**b**) PLA between RIPK1 and CASP8 in HT1080 cells on treatment with zVAD (i) or TNF/SM/zVAD (ii). (**c**) PLA between RIPK1 and CASP8 in SWISS-3T3 cells on the indicated treatments. Nuclei are stained with DAPI (4,6-diamidino-2-phenylindole; blue), PLA signals are in green and Phalloidin staining is in red.

RIPK1 can form protein complexes in the cytosol with CASP8, cFLIP (cellular FLICE-like inhibitory protein) and Fas-associated protein with a death domain (FADD). Depending on the signals that stimulate the assembly of RIPK1-based protein complexes, this complex is referred to as complex-II or ripoptosome^33^,^34^,^35^,^36^,^37^. Active CASP8 within complex-II/ripoptosome cleaves and inactivates RIPK1, thereby counteracting the assembly and stability of this complex^38^,^39^. The complex-II/ripoptosome is further negatively regulated by the E3 ubiquitin-ligases cIAP1, cIAP2 and XIAP, which target this complex for Ub-dependent inactivation^33^. When IAPs and/or CASP8 are inhibited, complex-II/ripoptosome complexes significantly accumulate and are stabilized^40^. Consistent with the notion that PLA can be adapted to detect complex-II/ripoptosome *in situ*, we found that specific primary antibodies against RIPK1 and CASP8 resulted in RIPK1/CASP8 proximity signals (Fig. 7a–i). Importantly, these proximity signals were significantly enhanced when IAPs and/or CASP8 were inhibited, such as following treatment with pharmacological inhibitors of IAPs (second mitochondria-derived activator of caspase mimetic, SM) (Fig. 7a(ii)) and stable expression of the viral CASP8 inhibitor CrmA (Fig. 7a(iii)). This is entirely consistent with co-immunoprecipitation experiments, demonstrating that the ripoptosome is stabilized following treatment with SM or CASP8 inhibitors^33^,^34^. The notion that the proximity technique reliably detects complex-II/ripoptosome formation *in situ* is further corroborated by the observation that treatment with tumour necrosis factor (TNF)/SM, which drives RIPK1-dependent formation of complex-II (ref. 35), resulted in a dramatic increase in RIPK1/CASP8 proximity signals within a few hours (Fig. 7b).

TNF is a pleiotropic cytokine inducing a variety of cellular responses ranging from inflammatory cytokine production, cell survival, cell proliferation and, paradoxically, CASP8-dependent cell death^41^,^42^. Although it is clear that many human pathogeneses are caused by deregulated TNF signalling, recent evidence suggests that some TNF-driven diseases might not only be due to aberrant TNF-mediated activation of nuclear factor-κB and the production of cytokines^43^, but also might be the result of deregulated TNF-induced cell death^44^. In this respect, it is interesting to note that TNF contributes to the neurotoxicity observed in retinal neurodegenerative disorders^45^. Given that mutations in MYO7A sensitize cells to apoptosis and retinitis pigmentosa^31^,^46^, and that MYO7A can interact with CASP8 in a RIPK1-dependent manner (Fig. 6d(iv)), we assessed whether MYO7A modulates TNF-dependent formation of complex-II/ripoptosome. Intriguingly, we found that RNAi-mediated knockdown of MYO7A enhanced TNF-induced formation of RIPK1/CASP8 complexes (Fig. 7c and compare Fig. 7c(iii) with Fig. 7c(iv)).

### Loss of MYO7A sensitizes cells to cytokine-induced cell death in a CASP8-dependent manner

**Figure 8:**
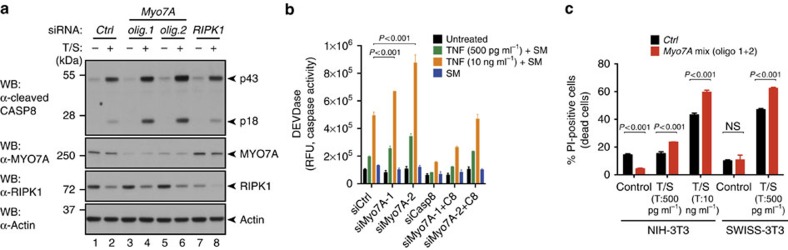
Loss of MYO7A sensitizes cells to cytokine-induced cell death in a CASP8-dependent manner. (**a**) WB analysis of activated CASP8 (p43 and p18 cleavage products), MYO7A, RIPK1 or ACTIN following the indicated treatments in NIH-3T3 cells. Depicted are representative westernblot images of three biological repeats. (**b**) DEVDase assays from lysates of NIH-3T3 cells subjected to siRNA targeting *Myo7A* or *Casp8*, and exposed to the indicated treatments. (**c**) FACS analysis of PI-positive NIH-3T3 or SWISS-3T3 cells subject to siRNA knockdown of the indicated genes followed by the indicated treatments. Error bars represent s.d. *P*-values are indicated. Experiments were conducted in triplicates.

Consistent with the notion that depletion of MYO7A caused enhanced formation of RIPK1/CASP8 complexes, we found that this was accompanied with enhanced processing and activation of CASP8, elevated levels of effector caspase activity (DEVDase) and reduced cell viability (Fig. 8a–c and Supplementary Fig. 4). Under the same conditions, knockdown of *Casp8* or *Ripk1* suppressed caspase activation (Fig. 8 and Supplementary Fig. 4), demonstrating that the enhanced cytotoxic effect of TNF on depletion of MYO7A is RIPK1 and CASP8 dependent. In all cell lines tested, knockdown of MYO7A enhanced TNF-induced caspase activation and cell death (Supplementary Fig. 4), extending this observation to other cell types. Therefore, these data indicate that MYO7A contributes to the proper regulation of CASP8, protecting cells from the cytotoxic effects of cytokines such as TNF. The observation that MYO7A binds to the DED of CASP8 (Fig. 6b) is particularly intriguing, because for full activation CASP8 requires sequential interaction of CASP8 molecules, via their DED domains, to form a caspase-activating chain^47^. As MYO7A binds to the DED of CASP8 and dampens its activation, it is possible that MYO7A suppresses the formation of CASP8 chains, thereby acting as a chain terminator. Alternatively, MYO7A might regulate the stability/turnover of complex-II/ripoptosome. A characteristic feature of complex-II/ripoptosome is that it regulates its own stability^38^,^39^. Once RIPK1 adopts a binding-competent state, it recruits FADD, CASP8 and cFLIP_L_. FLIP_L_-mediated activation of CASP8 results in cleavage of RIPK1, which results in disassembly of the complex. Given that depletion of MYO7A results in enhanced complex formation (Fig. 7c(iv)), it is possible that the presence of MYO7A favours CASP8-mediated cleavage of RIPK1 and disassembly of the complex. This would be reminiscent to the situation in *Drosophila* where CK helps DRONC to drive the cleavage of SGG46. In the absence of MYO7A, less RIPK1 would be cleaved, which results in a build-up of complex-II/ripoptosome formation (as evidenced in Fig. 7c(iv)), which in turn would lead to enhanced caspase activation. According to this scenario, MYO7A would not actually change the activity of CASP8 but alter the stability of complex-II/ripoptosome. These two models are not mutually exclusive, as both mechanisms could operate simultaneously. Ultimately, quantitative and structural mass spectrometric approaches will be necessary to determine the stoichiometry of RIPK1, FADD, CASP8 and FLIP_L_ in the presence and absence of MYO7A.

## Discussion

**Figure 9:**
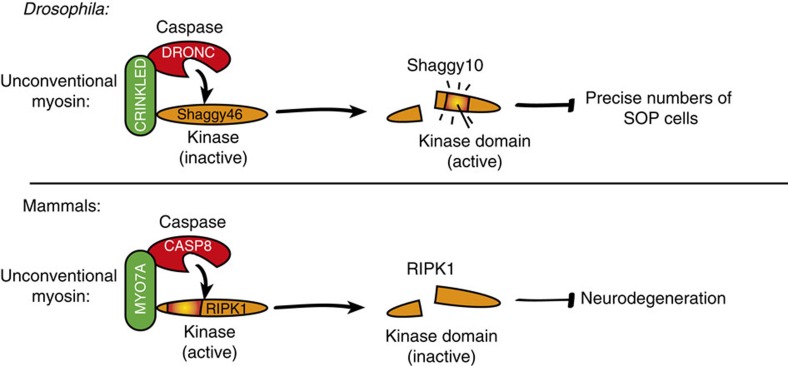
Schematic model depicting the evolutionary conserved and unifying principle of caspase-mediated regulation of kinases. In *Drosophila*, binding of CK to DRONC favours DRONC-mediated cleavage and maturation of SGG46 into its active form SGG10. In mammals, MYO7A might regulate CASP8-mediated cleavage of RIPK1, which results in disassembly of the ripoptosome complex. In the absence of MYO7A, less RIPK1 would be cleaved, which results in a build-up of complex-II/ripoptosome formation, which in turn would lead to enhanced caspase activation.

Usher syndrome, the leading cause of hereditary combined hearing and vision loss, is characterized by sensorineural deafness and progressive retinal degeneration^7^. Mutations in several different genes produce Usher syndrome; however, the proximal cause of sensory cell death remains unknown. Our finding that MYO7A interacts with the initiator CASP8 and dampens its activation may help to explain why patients with mutations in MYO7A suffer progressive loss of sensory neurons. Given that RIPK1 and CASP8 take part in the defense of homeostasis downstream of many cytokine receptors, it is possible that inflammatory signals such as TNF contribute to the onset and progression of retinitis pigmentosa due to aberrant activation of RIPK1-dependent cell death. Our findings have important implications, as they suggest that anti-inflammatory biologics, such as Enbrel (a TNF blocker), might help to delay or even prevent the onset of this debilitating disease. Although CASP8 can initiate apoptosis downstream of TNF death receptors, the normal physiological function of CASP8 is to transmit a pro-survival signal that suppresses RIPK1-dependent necroptosis. Suppression of RIPK1-dependent necroptosis by CASP8 requires its catalytic activity. One of the key substrates processed by CASP8 is RIPK1. This destabilizes RIPK1-based death complexes^38^,^39^. The observation that RIPK1-based platforms accumulate on depletion of MYO7A might indicate that CASP8-mediated cleavage and inactivation of RIPK1 is less effective in the absence of MYO7A. This is highly reminiscent of the situation in *Drosophila*, where CK assists DRONC in cleaving the kinase SGG46. Thus, in both *Drosophila* and mammals, CK and MYO7A appear to take part in caspase-mediated regulation of kinases, revealing a unifying principle of caspase-mediated regulation of cellular processes (Fig. 9).

## Methods

### Reagents

Constructs were generated by PCR and cloned into pAc, pMT, pcDNA3 (Invitrogen) or pEF6 and verified by sequencing. Specific point mutations were generated using site-directed mutagenesis with Pfu Turbo polymerase (Stratagene). The *Ub-sgg10* fusion construct was generated by PCR using Easy-A polymerase and cloned into pMT-V5/His. The following antibodies were used: α-MYC (Sigma, M5546, 1:2,000), α-HA (Roche, 11867423001, 1:2,000), α-V5 (Serotec, MCA1360, 1:2,000), α-RIPK1 (BD Biosciences, 610459,1:1,000 for western blotting (WB) and 1:50 for immunofluorescence studies), α-Actin (Santa Cruz, sc-1615, 1:4,000), α-MYO7A (Developmental Studies Hybridoma Bank, 138-1-s, 1:1,000 for WB and 1:50 for immunofluorescence studies), α-CASP8 for WB (MBL, M032-3, 1:5,000), α-CASP8 to detect cleaved CASP8 (for human: R&D, AF1650, 1:2,000; for mouse: Cell Signaling Technology, 9429, 1:1,000), rabbit and goat α-CASP8 (Santa Cruz Biotechnology, sc-7890 and sc-6136, both 1:50 for immunofluorescence studies), CF488A-donkey α-mouse IgG (Biotium, A21202, 1:1,000), CF633-donkey α-rabbit IgG (Biotium, 20125, 1:1,000) and CF633-donkey α-goat IgG (Biotium, 20127, 1:1,000). SM (SM164) was a gift from Shaomeng Wang (University of Michigan) and TNF was obtained from Enzo Life Sciences. Uncropped WBs are shown in Supplementary Fig. 5.

### Fly stocks

Flies were raised at 25 °C according to standard procedures, unless stated otherwise. RNAi stocks were obtained from the Vienna *Drosophila* RNAi Center, with the exception of *GFP* RNAi (a gift from R. Ueda) and *LacZ* RNAi (a gift from M. Miura). *UAS-GFP-ck*^*TAIL*^ was a gift from D. Kiehart. *UAS-DmIKKɛ*^*G250D*^ (DN-DmIKKɛ) and *UAS-sgg46*^*D235G/D300G*^ were gifts from M. Miura. *GMR-hid*,ey-*FLP* were previously described^18^. All other stocks were obtained from the Bloomington *Drosophila* Stock Center or described previously.

### Quantification of macrochaete

Crosses were performed at 18 °C. The macrochaete on the scutellum and the central portion of the notum were analysed. Any macrochaete that were present in addition to the usual numbers (four on the scutellum and four on the central part of the notum) were scored as ectopic macrochaete.

### Border cell migration

Ovaries were dissected in PBS, fixed in 4% formaldehyde for 20 min and permeabilized in PBS containing 0.1% Tween 20. Ovaries were stained with 4,6-diamidino-2-phenylindole and phalloidin (Invitrogen). After mounting in Vectashield Hardset Mounting Medium (Vector Laboratories), confocal images of stage-10 egg chambers were taken. The distance the border cells had moved was measured using ImageJ software in at least 30 stage-10 egg chambers and expressed as a percentage of the total distance from the anterior pole to the oocyte.

### Tissue culture and confocal microscopy

*Drosophila* S2 cells and mammalian SKOV3, HT1080, NIH3T3 and Swiss3T3 were obtained from ATCC. SKOV3^CrmA^ were described in ref. 33. Small interfering RNA (siRNA) oligos are as follows: human *Ripk1* siRNA: 5′- CCAACUAUCUAGGAAAUACtt -3′; human *Myo7A*: 5′- GGACGGGUGUGUACUUUGU -3′; non-targeting: AllStars Negative control-1027281 (Qiagen) (for mouse and human); mouse *Myo7A-1*: (Qiagen) Mm_Myo7a_1 FlexiTube siRNA, Cat:SI01322279; mouse *Myo7A*: (Qiagen) Mm_Myo7a_2 FlexiTube siRNA, Cat:SI01322286; mouse *Ripk1*: 4390771-ID:s72977 (Ambion); and mouse *Casp8* siGenome SMART pool (Thermo Scientific), Cat M-043044-01. siRNA oligos were transfected using HiPerFect (Qiagen) 48 h before experimental conditions. *Drosophila* S2 cells were transfected with Effectene (Qiagen) according to the manufacturer's instructions. For immunofluorescence staining of mammalian cells, 10^5^ cells were plated on 13 mm glass cover slips (VWR) and retro-transfected with 50 nmol siRNA as indicated. Cells were then treated with the indicated biologics and fixed in 4% paraformaldehyde for 10 min. Following 10 min permeabilization with PBS containing 0.5% Triton X-100, cells were incubated for 1 h in 5% BSA in PBS. Respective primary antibodies were then added as follows: α-RIPK1 (BD Bioscience), rabbit or goat α-CASP8 (Santa Cruz Biotechnology) and α-MYO7A (Sigma). Alexa Fluor 633-conjugated phalloidin (Invitrogen) was used to stain actin filaments. Secondary fluorescent conjugated antibodies were then added as follows: 1:1,000 CF488A-donkey α-mouse and 1:1,000 CF633-donkey α-rabbit or 1:1,000 CF633-donkey α-rabbit. Cells were visualized by confocal microscopy (objective × 40, Zeiss LSM710). For Fig. 4c,d, SM was used at 100 nM and SKOV3 cells were treated for 14–16 h. For Fig. 4f–i, TNF (Enzo Life Sciences) was used at 10 ng ml^−1^ (unless stated otherwise) in conjunction with 10 nM SM and cells were treated for 8 h. Expression of CrmA was induced using 100 nM Tamoxifen, at least 4 h before analysis.

### Cell lysis and immunoprecipitation

Cells were lysed in 50 mM Tris pH 7.5, 150 mM NaCl, 0.1% Triton X-100, 10% glycerol and 1 mM EDTA. Where lysates were used for immunoprecipitations, complete protease inhibitor cocktail (Roche) was added. Immunoprecipitations were performed using α-Myc or α-HA-agarose beads (Sigma). Samples were examined by immunoblot analysis using either chemiluminescence (Amersham Biosciences) or Odyssey Infrared Technology (LI-COR Biosciences).

### Proximity ligation assay

PLA was performed according to the manufacturer's protocol using the Duolink Detection Kit (Cambridge BioScience Ltd). Immunofluorescence staining of RIPK1 and CASP8 for the Duolink was carried out following the above-described protocol for immunofluorescence detection up until the primary antibody incubation step. Probe incubation, ligation and amplification reaction were carried out according to the manufacturer instructions. Cy3 signal amplification was used for the assay. Cells were examined with a confocal microscope (objective × 40, Zeiss LSM 710).

### FACS and DEVDase assays

For FACS and DEVDase assays, 10^4^ cells were plated in a 96-well plate and reverse siRNA transfection was performed with 50 nM total siRNA for 40 h (25 nM of each individual siRNA balanced with control siRNA). Cells were treated with TNF (500 pg ml^−1^ or 10 ng ml^−1^) plus SM in 150 μl for 24 h (FACS) or 5–6 h (DEVDase). For FACS analysis, medium containing dead cells was transferred to a round-bottom 96-well plate, live cells were trypsinized in 50 μl, harvested with 100 μl of medium containing 1 μg ml^−1^ propidum iodide (Sigma) and combined with dead cells (total volume 300 μl). The 96-well plate was analysed by FACS using a plate reader. Data shown are from 5,000 cells per condition. For DEVDase assays, the medium was removed after treatment and 20 μl 1% Triton lysis buffer was added to each well. Plates were placed at −80 °C to aid cell lysis. Plates were thawed at room temperature for 15 min, after which 180 μl DEVDase assay mix (20 μM Ac-DEVD-AMC (Sigma), 1 mM dithiothreitol, 50 mM Tris pH 7.5, 150 mM NaCl, 0.1% Triton X-100 and 5% glycerol) was added to each well (it is noteworthy that cell lysates were not cleared). Reactions were incubated at room temperature for up to 24 h. DEVDase activity was read at 380 nM excitation/460 nM emission.

### Statistical analysis

For most experiments, data were analysed pairwise with two-tailed *t*-tests assuming equal variance, using the following website: http://studentsttest.com. Macrochaete data were analysed with Fisher's exact test, using the following website: http://www.quantitativeskills.com/sisa/statistics/fiveby2.htm.

## Additional information

**How to cite this article:** Orme, M. H. *et al*. The unconventional myosin CRINKLED and its mammalian orthologue MYO7A regulate caspases in their signalling roles. *Nat. Commun.* 7:10972 doi: 10.1038/ncomms10972 (2016).

## Supplementary Material

Supplementary Information Supplementary Figures 1-5, Supplementary Methods and Supplementary References

Supplementary Data Set 1 Mass Spec Data Set
