# Supplementary material for: The unconventional myosin CRINKLED and its mammalian orthologue MYO7A regulate caspases in their signalling roles
Source: Nat Commun. 2016 Mar 10;7:10972. doi: 10.1038/ncomms10972 (PMC4792956; doi:10.1038/ncomms10972)
Supplement: Supplementary Information — Supplementary Figures 1-5, Supplementary Methods and Supplementary References [file ncomms10972-s1.pdf]

Supplementary Figure 1

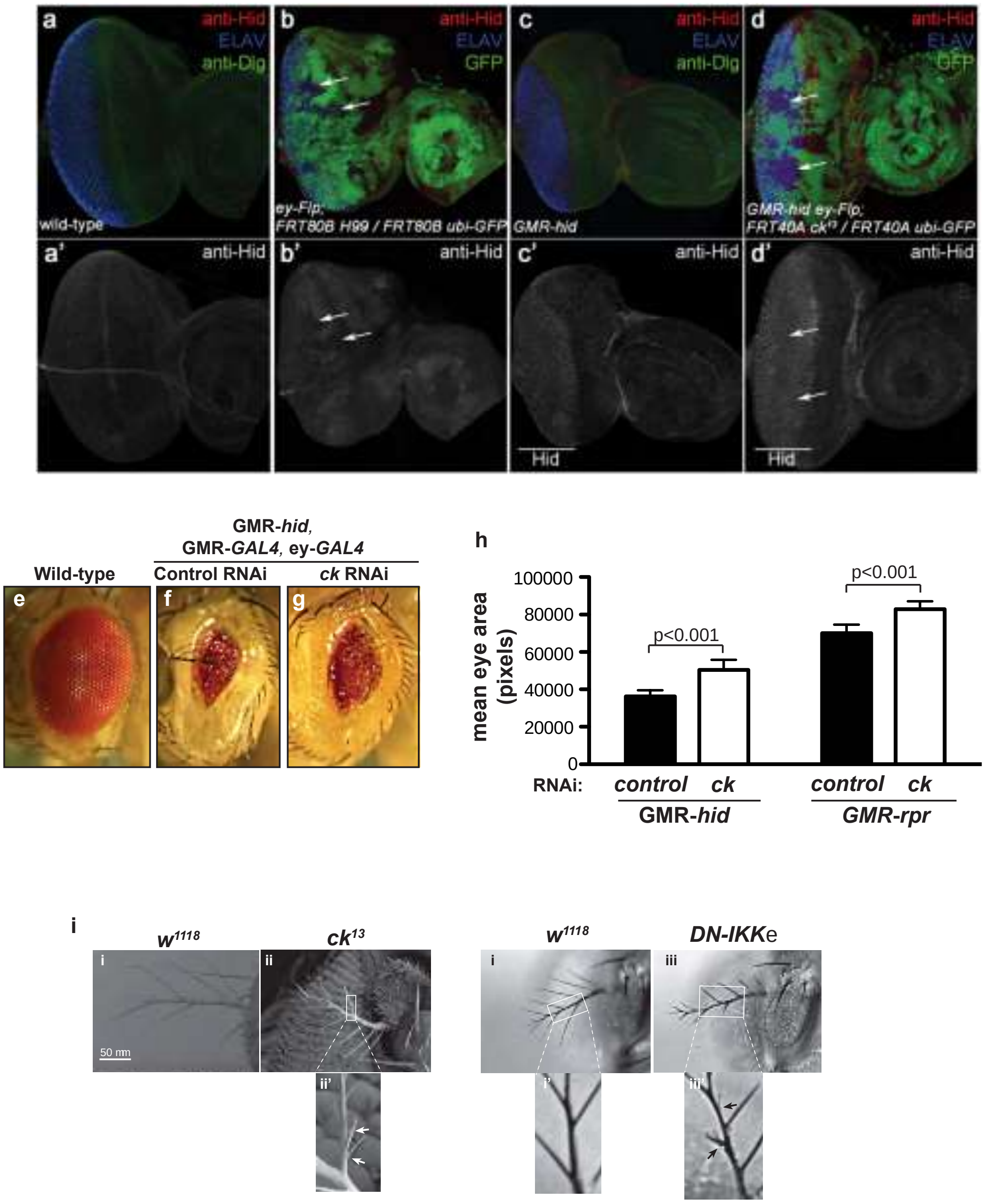

Supplementary Figure 1. Genetic loss or RNAi-mediated depletion of ck ameliorates the eye phenotypes induced by expression of IAP-antagonists, without affecting the HID protein levels in *GMR-hid*. **(a-d)** *ck*^{13} mutants do not affect HID protein levels in *GMR-hid*. Shown are eye-antennal imaginal discs from wandering 3rd instar larvae. ELAV labels photoreceptor neurons in the posterior half of the eye imaginal disc and overlaps with the *GMR* expression domain driving *hid*. DLG labelling marks the outline of the discs. Lower panels show the HID channel only (grey). Posterior is to the left. Genotypes are indicated in each panel. (a,a') Wild-type control disc labeled for HID (red), ELAV (blue) and DLG (green) proteins. Endogenous HID levels are homogenous. (b,b') *H99* mosaic control disc labelled

with α-HID (red) and α-ELAV (blue) antibodies. *H99* mutant clones, in which the *hid* gene is deleted, are marked by the absence of GFP. The arrows highlight two example clones, which do not label with the α-HID antibody (lower panel) demonstrating specificity of the antibody. (c,c') *GMR-hid* eye disc labelled with α-HID, α-DLG and ELAV antibodies. HID protein levels are elevated in the posterior part of the eye disc due to *GMR* expression (c') and overlaps with ELAV (c). The bar in (c') marks the extent of *hid* expression by *GMR*. (d,d') *GMR-hid* eye disc mosaic for *ck* labelled with α-HID and α-ELAV antibodies. *ck* clones are marked by the absence of GFP. HID protein levels in the GMR domain are unchanged in *ck* mutant clones (d'). The bar in (d') marks the extent of hid expression by *GMR*. **(e)** Wild-type eye. **(f,g)** dsRNA of *GFP* or *ck* was expressed in the eye using the drivers *ey-Gal4* and *GMR-GAL4*, and its effect on *GMR-hid*-mediated eye phenotypes analysed by light microscopy of whole mounts. Two drivers were used to knockdown the target gene before and during expression of *rpr* or *grim.* The *ey driver* allows expression of the RNAi transgene early on in eye development, while the *GMR driver* induces expression of *rpr/hid* at a later stage (after the morphogenic furrow). Therefore, *ey-Gal4* was used to drive early expression of the RNAi to knock down the levels of protein before expression of *rpr/hid* begins. *GMR-Gal4* was additionally used to ensure continued expression of the RNAi transgene during the period of *rpr/hid* expression. **(h)** The graph depicts the eye size of flies expressing *hid* in the eye, together with dsRNAi against *gfp* as a control or *ck* (left side), and flies expressing *rpr* in the eye, together with dsRNAi against *white* as a control or *ck* (right side). Shown is the mean eye size of at least 12 animals (with the exception of the *rpr* control, which represents six animals) ±SE. Genotypes**:** **(f)** *GMR-hid, GMR-Gal4/ UAS-GFP-IR; ey*-Gal4/ + **(g)** *GMR-hid, GMR-Gal4/ +; UAS-ck-IR/ ey-Gal4* **(h)** First bar: as (f) [n=9]. Second bar: as (g) [n=12]. Third bar: *ey-Gal4/ +; UAS-w-IR/ GMR-rpr, GMR-Gal4* [n=3]. Fourth bar: *ey-Gal4/ +; UAS-ck-IR/ GMR-rpr, GMR-Gal4* [n=13]. Error bars represent standard deviation. p values are less than 0.001 with respect to the control. P values were calculated using an unpaired, two-tailed t-test. **(i)** SEM pictures and light micrographs of representative arista from w^{1118} (i-i and i-i'), ck^{13}(i-ii and i-ii') and *Ap-Gal4/ UAS-DmIKK*ε^{G250D} (i-iii and i-iii') flies (also see [1, 2]). Extra branches are indicated by arrowheads.

Supplementary Figure 2

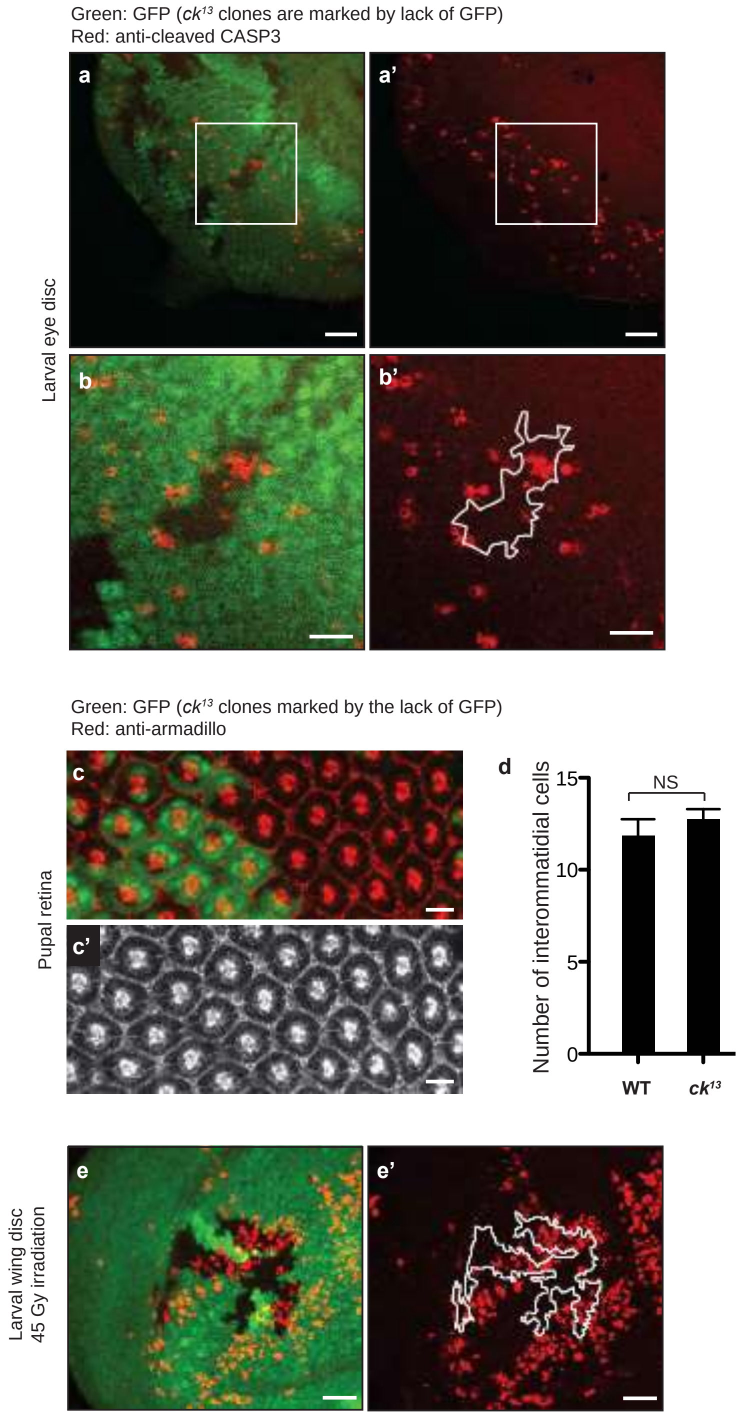

Supplementary Figure 2. CK is not a rate-limiting factor during developmental cell death in the eye, or execution of cell death in response to high doses of irradiation. **(a-e)** Confocal microscope images, with *ck*^{13} clones marked by the absence of GFP (green). **(a, b)** An eye disc from a third instar larva, stained with an antibody against cleaved CASP3 (red) detecting developmental apoptosis. The white box depicts the area of magnification shown in b and b’. Clone boundaries are indicated by the white

lines in (b’). Note, cleaved CASP3 staining does not respect the clone boundaries. Scale bars represent 25 μm in (a) and (a’), and 10 μm in (b) and (b’) Genotype: *yw, hs-Flp/+* or *Y;ck^{13},FRT40A/P[ubi-GFP], FRT40A*. **(c** and **c’)** A retina at 40 hours after puparium formation, stained with an antibody against armadillo (red in (c), grey in (c’)) to outline cells. Scale bars represent 10 μm. Genotype: *yw, ey-Flp/ +* [n=8] *or Y; ck^{13}, FRT40A/ P[ubi-GFP], FRT40A* [n=12] **(d)** Quantification of the number of interommatidial cells per ommatidial unit. There is no significant difference in the number of interommatidial cells in wild type or mutant tissue. Error bars represent standard error of the means. p values are less than 0.38 (not significant (NS)) with respect to the control. P values were calculated using an unpaired, two-tailed t-test. Genotype *yw, ey-Flp/ + [n=8] or Y; ck^{13}, FRT40A/ P[ubi-GFP], FRT40A* [n=12]. **(e)** A wing disc from an X-irradiated third instar larva, stained with an antibody against cleaved CASP3 (red). The clone boundary is indicated by a white line in (e’). Scale bars represent 25 μm. Genotype: *yw, hs-Flp/+* [n=8] or *Y;ck^{13},FRT40A/P[ubi-GFP], FRT40A* [n=12].

Supplementary Figure 3

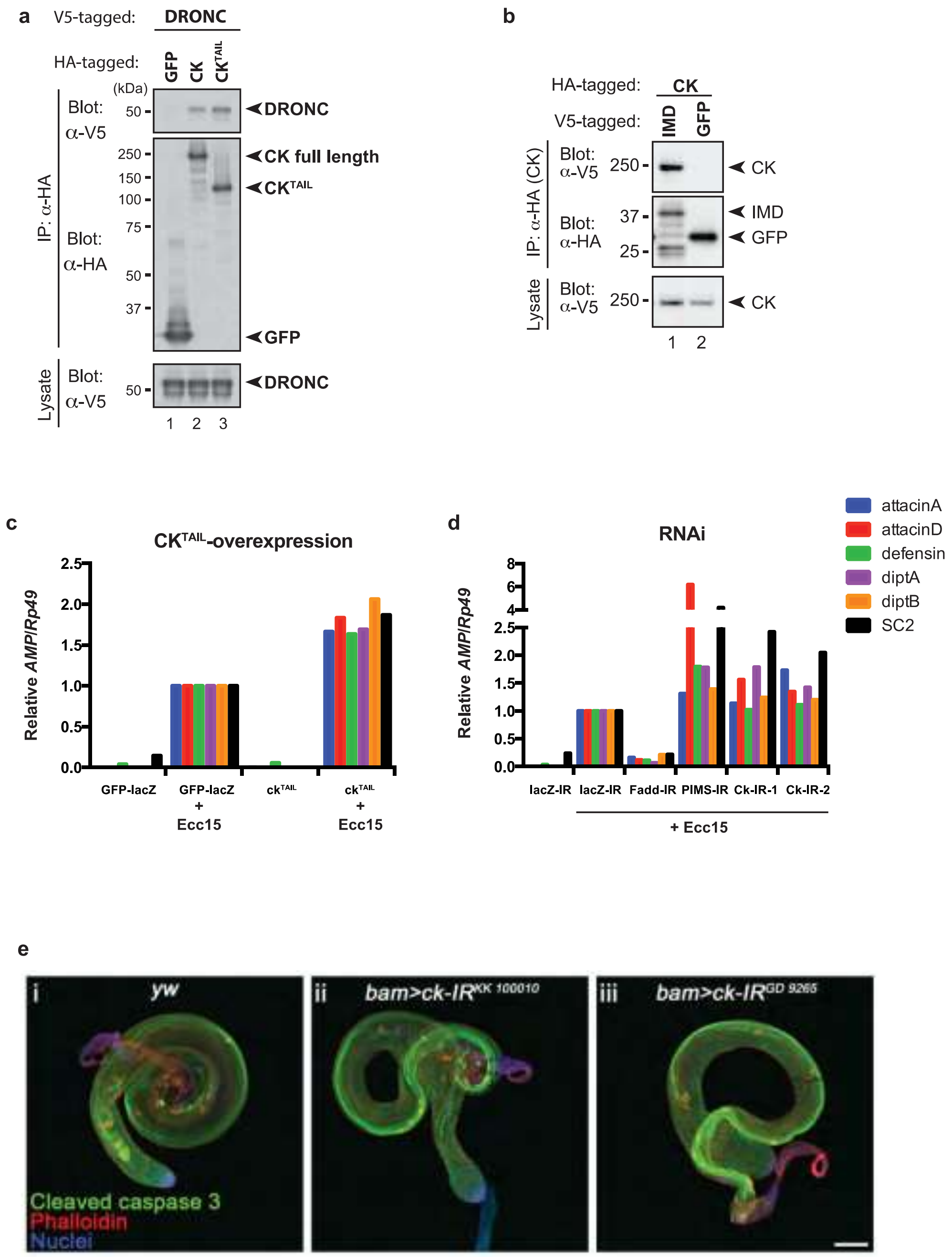

Supplementary Figure 3. (a) CK^{TAIL} binds to DRONC as efficiently as wild-type CK. V5-tagged DRONC was co-expressed with HA-GFP, HA-CK or HA-CK^{TAIL}. α-HA immunoprecipitation was performed and binding to CK was determined by immunoblotting. **(b-d)** CK binds to IMD, and acts as a weak negative regulator of IMD signalling. (b) HA-tagged CK was co-expressed with V5-GFP or V5-IMD. α-HA immunoprecipitation was performed and binding to CK was determined by immunoblotting. (c) Fat body specific expression (*C564-Gal4*) of CK^{TAIL}, but not the control protein GFP-LacZ, enhances the induction of anti-microbial peptide genes

(AMPs) in response to septic injury with the Gram-negative bacteria *Ecc15*. Quantitative RT-qPCR analysis of the indicated AMPs expression is shown. Rp49 was used as the experimental expression standard. Show is the data of a pool of 30 flies (2-5 days old). (d) Consistent with an earlier report[3], we find that fat body-specific (*C564-Gal4*) knockdown of *ck* results in enhanced induction of AMPs following septic injury with *Ecc15*. AMP expression was evaluated as indicated above. (e) Knockdown of *ck* does not affect caspase activation in spermatids. The individualization complex (IC) is in red (phalloidin labels the IC), nuclei in blue, and active caspases in green. The advancing cystic bulge (CB) collects the spermatids' cytoplasm and most of the organelles and eventually is pinching off from the base of the cyst and discarded as a waste bag (WB). Shown is the result from two independent *ck* RNAi lines. *Bam-Gal4* is used to drive expression of the *ck* RNAi transgene specifically in the testis. Two different *ck* RNAi lines from VDRC were used (ii; KK 100010 and iii; GD 9265).

Supplementary Figure 4

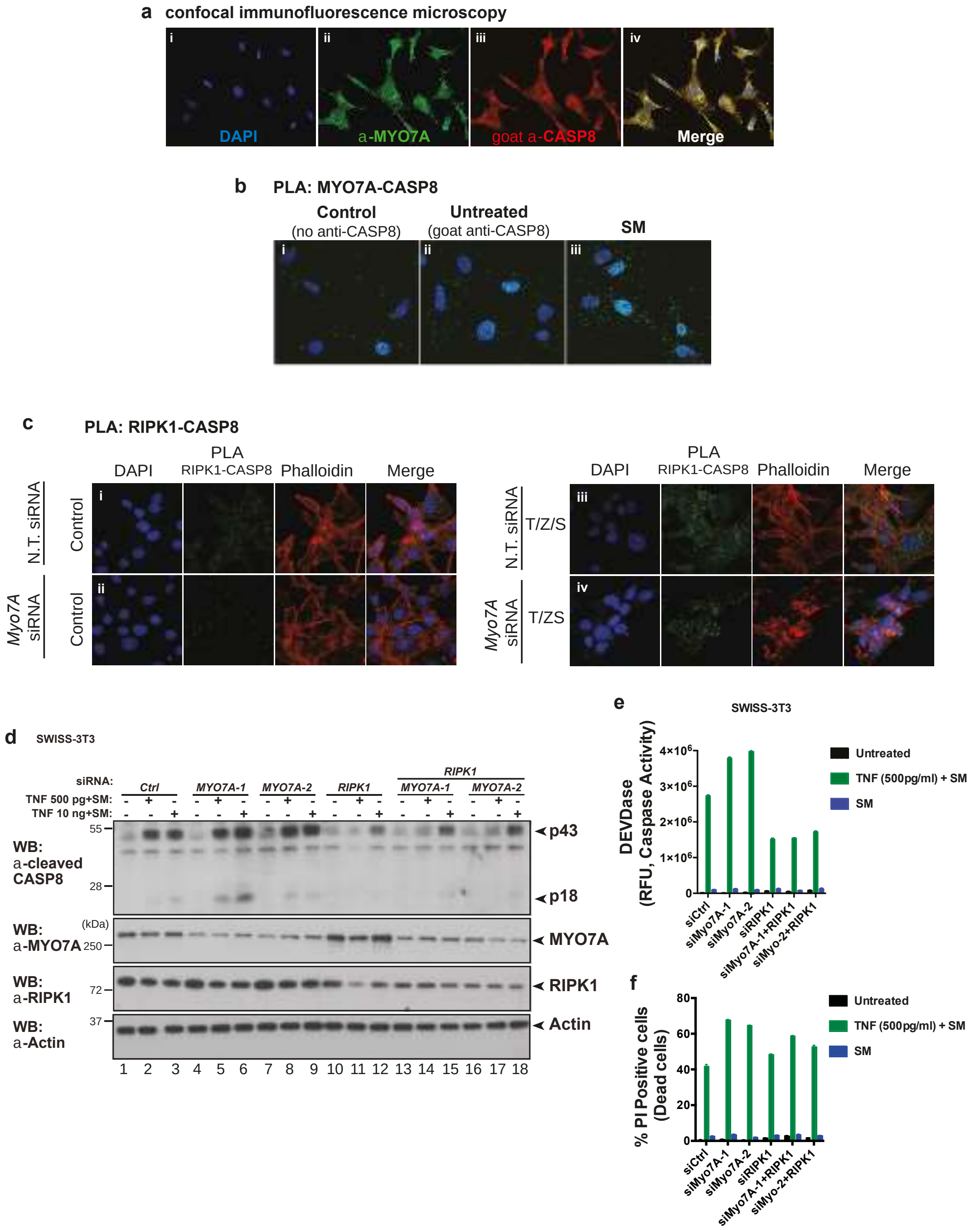

Supplementary Figure 4. Loss of MYO7A sensitizes cells to cytokine-induced cell death in a CASP8-dependent manner. **(a)** Confocal microscopy images showing co-localization of MYO7A (green) and CASP8 using goat α-CASP8 (red) in SKOV3 cells. Nuclei stained with DAPI (blue). **(b)** Representative confocal microscopy images of PLA using mouse α-MYO7A and either no α-CASP8 antibodies (i) or goat α-CASP8 antibodies (panels ii-iii) in SKOV3 cells. PLA signals are in green, and

nuclei are stained with DAPI (blue). PLA between MYO7A and CASP8 upon treatment with 100 mM SM for 5 hours (iii). **(c)** PLA between RIPK1 and CASP8 in NIH-3T3 cells upon the indicated treatments. Nuclei are stained with DAPI (blue), PLA signals are in green, and phalloidin staining is in red. **(d)** Western blot analysis of activated CASP8 (p43 and p18 cleavage products), MYO7A, RIPK1 or Actin following the indicated treatments in SWISS-3T3 cells. **(e)** DEVDase assays from lysates of SWISS-3T3 cells subjected to siRNA targeting *Myo7A* or *Casp8*, and exposed to the indicated treatments. **(f)** FACS analysis of PI positive SWISS-3T3 cells subject to siRNA knockdown of the indicated genes followed by the indicated treatments. Error bars represent standard deviations.

Supplementary Figure 5. Uncropped immunoblots.

Fig.1a

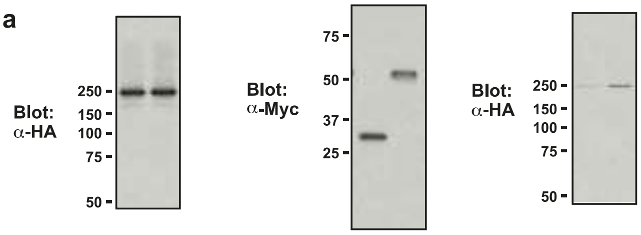

Fig.1b

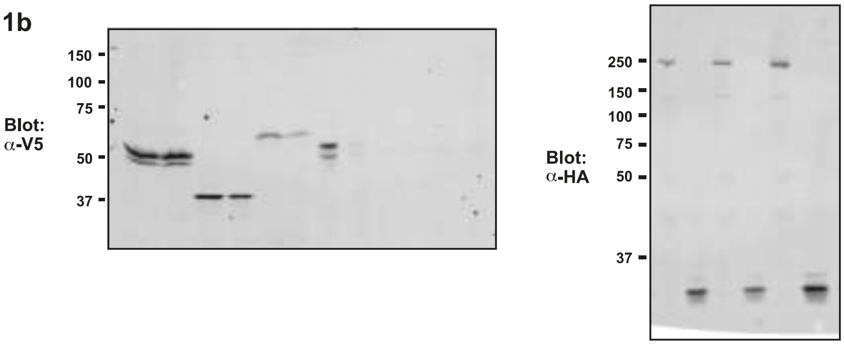

Fig.2f

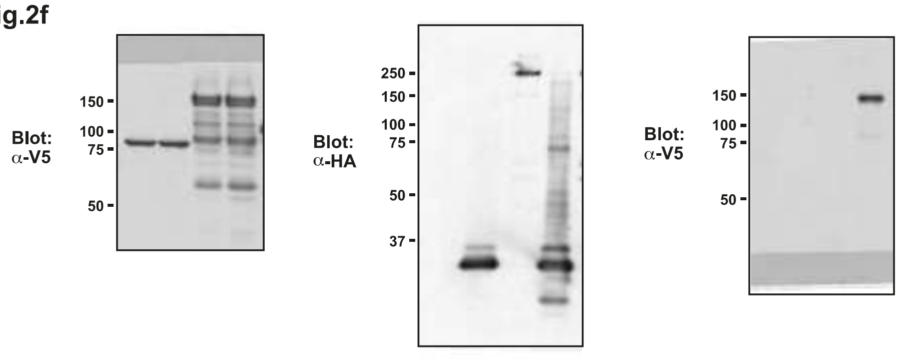

Fig.2g

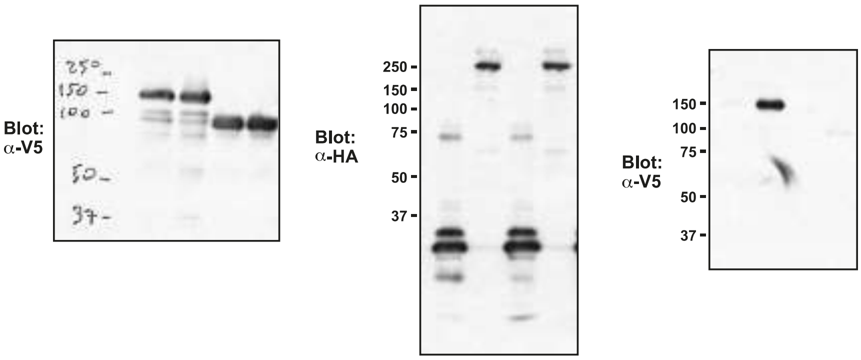

Fig. 4a

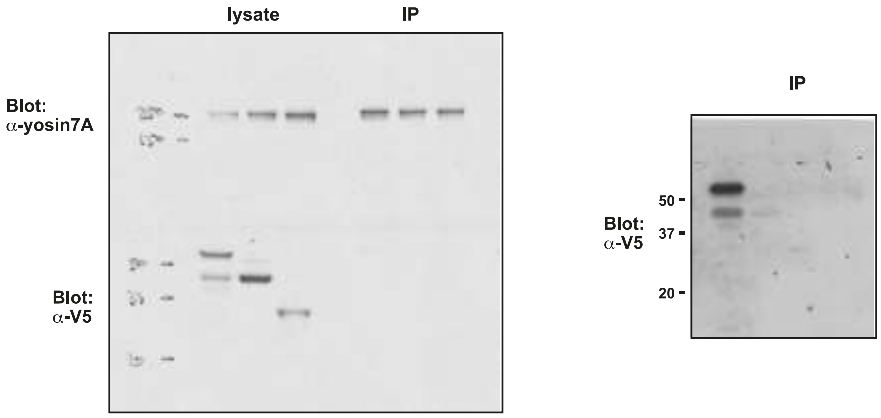

Suppl. Fig. 4d

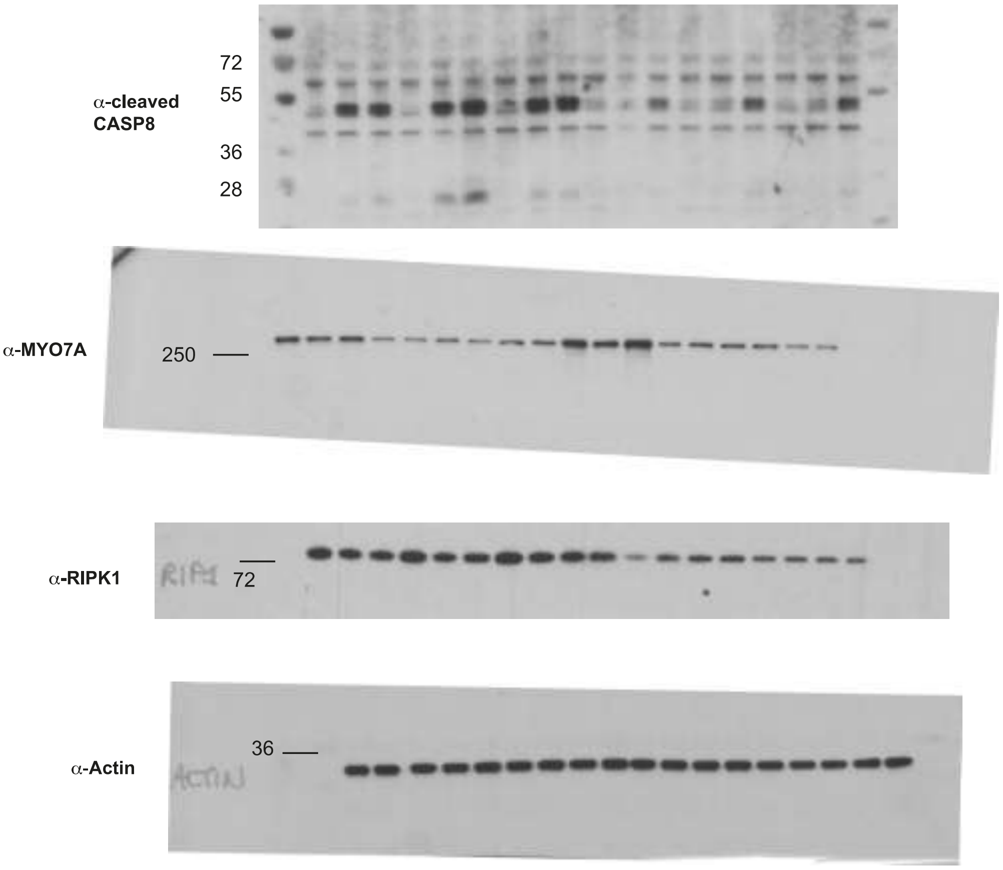

Fig.4g

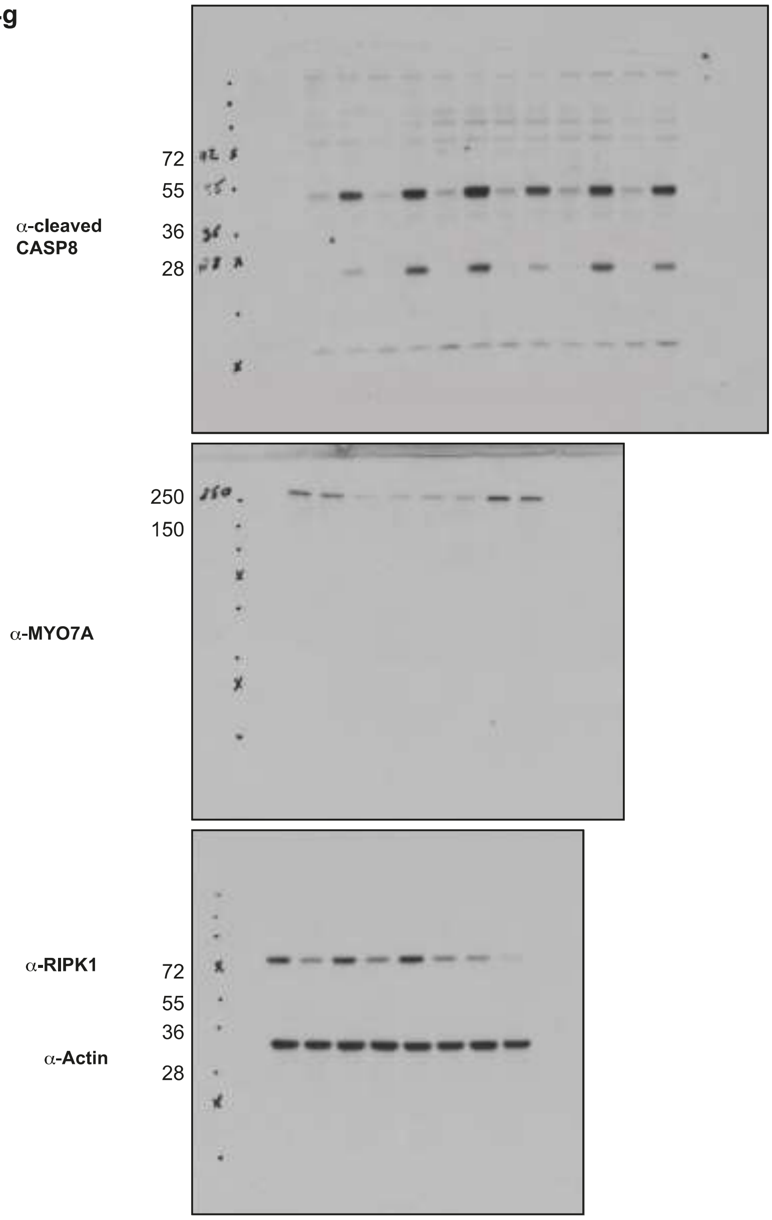

α-cleaved CASP8
72
55
36
28
250
150
α-MYO7A
α-RIPK1
72
55
36
α-Actin
28

Supplementary Methods

Antibodies. The following antibodies were used: α-cleaved-*Drosophila* DCP1 antibody Asp 216; Cell Signaling Technology, 1:50), α-DLG (Mouse, Developmental Studies Hybridoma Bank, 4F3, 1:200), α-HID (Santa Cruz; d300, 1:200), α-Armadillo (Developmental Studies Hybridoma Bank, N27A1, 1:100).

Infection experiments. Adult flies were injected using the Nanoject II (Drummond Scientific). 13,6 nL of cultured *Erwinia carotovora* subsp. *carotovora* 15 (*Ecc15*) bacterial solution with an optical density of 0.3 was injected through the lateral part of the thorax. Following septic injury, flies were incubated at 25°C, and were lysed for qRT-PCR. 20 flies were used for each genotype or experimental condition.

Quantitative RT-PCR. mRNA from whole flies was extracted using the RNeasy Mini kit (QIAGEN) and cDNAs were synthesized with the Quantitech Reverse Transcription kit (QIAGEN). The quantitative PCR reaction was performed with the MesaBlue qPCR mastermix Plus for SYBR green (Eurogentech) using the 7900HT RT-PCR machine. AMP mRNA amount was normalized to the endogenous control RP49, and data were analyzed with the SDS program v2.2.1 (Applied Biosystems). qPCR primers were: *Ribosomal protein L32* (Rp49): GACGCTTCAAGGGACAGTATCTG (forward), AAACGCGGTTCTGCATGAG (reverse); *attacin D*: GTCACTAGGGTTCCTCAG (forward), GCCGAAATCGGACTTG (reverse); *diptericin A*: GCTGCGCAATCGCTTCTACT (forward), TGGTGGAGTGGGCTTCATG (reverse); *diptericin B*: AGCCTGAACCACTGGCATA (forward), AGATCGAATCCTTGCTTTGG (reverse); *defensin*: GTTCTTCGTTCTCGTGG (forward), CTTTGAACCCCTTGGC (reverse); *attacin A*: CCCGGAGTGAAGGATG (forward), GTTGCTGTGCGTCAAG (reverse); *pgrp-sc2*: AACTACCTGAGCTACGCCGTGAT (forward), AGCAGAGGTGAGGGTGTTGGTATT (reverse); *pims*: GGCGTTCGTGTGATAG (forward), CTCAATGCGGTACTCC (reverse).

Spermatide individualisation experiment. The *bam-Gal4* line contains two copies of the driver, one copy on the X chromosome and one on the 3rd chromosome, as well as a single copy of *UAS-Dicer* on the 3rd chromosome. The line was generated by crossing lines obtained from L. Gilboa (WIS, Israel) and M. Wolfner (Cornell University, USA). Testes from young (0-2 day old) adults were dissected in testis

buffer, immediately moved into ice-cold fix solution (4% paraformaldehyde [PFA] diluted in PBS) within a glass well plate positioned on ice. Following the dissections, the fixated testes were incubated for 20 minutes at room temperature, rinsed three times for 10 minutes with PBX (PBS with 0.1% Triton-X), blocked with PBS/BSA (1% BSA in PBS) for 45-60 minutes at room temperature, and incubated overnight at 4°C with the primary antibody (rabbit polyclonal α-cleaved-*Drosophila* DCP1 antibody Asp 216; Cell Signaling Technology) diluted 1:50 in PBS/BSA. Testes were then rinsed in PBX, incubated with α-rabbit secondary antibody (Jackson ImmunoReaserch; 1:250) and Phalloidin-TRITC (used to label F-Actin, i.e. the IC; Sigma, 1:500) for one hour at room temperature, rinsed again and mounted in Vectashield mounting medium with DAPI (Vector laboratories).

Quantification of interommatidial cells. 40-hour-APF retinas were stained with an anti-Arm antibody to label the cell outlines. The IOCs for a single ommatidium were quantified by drawing a hexagon connecting the centres of the six surrounding neighbouring ommatidia. All IOCs within that hexagon were then counted. Cells straddling the boundary of the hexagon were counted as half a cell. For each genotype, the cells surrounding at least eight ommatidia were counted.

References:

1. Kiehart DP*, et al.* Drosophila crinkled, mutations of which disrupt morphogenesis and cause lethality, encodes fly myosin VIIA. *Genetics* **168**, 1337-1352 (2004).

2. Oshima K*, et al.* IKK epsilon regulates F actin assembly and interacts with Drosophila IAP1 in cellular morphogenesis. *Curr Biol* **16**, 1531-1537 (2006).

3. Ragab A, Buechling T, Gesellchen V, Spirohn K, Boettcher AL, Boutros M. Drosophila Ras/MAPK signalling regulates innate immune responses in immune and intestinal stem cells. *EMBO J* **30**, 1123-1136 (2011).
